# Increased risk of periodontitis in patients with psoriatic disease: a nationwide population-based retrospective cohort study

**DOI:** 10.7717/peerj.4064

**Published:** 2017-11-16

**Authors:** Ni-Yu Su, Jing-Yang Huang, Chien-Jen Hu, Hui-Chieh Yu, Yu-Chao Chang

**Affiliations:** 1 Department of Periodontics, Chung Shan Medical University Hospital, Taichung, Taiwan; 2 School of Dentistry, Chung Shan Medical University, Taichung, Taiwan; 3 Department of Medical Research, Chung Shan Medical University Hospital, Taichung, Taiwan

**Keywords:** Psoriasis, Periodontitis, Psoriatic arthritis, Nationwide population, Cohort study

## Abstract

**Aims:**

Periodontitis and psoriatic disease, including psoriasis (PS) and psoriatic arthritis (PsA), share the common risk factors and co-morbidities. However, the risk of periodontitis in patients with psoriatic disease still needs further investigation. This study was a nationwide population-based retrospective cohort study assessing the risk of periodontitis from psoriatic disease exposure.

**Materials and Methods:**

Patients with newly diagnosed psoriatic disease from 2003 to 2012 were identified from the Taiwanese National Health Insurance Research Database. The 1:4 ratio propensity score matched controls were selected from no psoriatic disease participations. The subsequent risk of periodontitis was evaluated in exposure and comparison groups. Multiple Cox proportional hazard models were used for the estimation.

**Results:**

A total of 3,487 psoriatic disease patients and 13,948 controls were identified. Incidence rate of periodontitis was higher in patients with PsA. The adjusted hazard ratio (aHRs) for moderate/severe periodontitis were 0.85 (95% CI [0.65–1.11]) in PS group and 1.66 (95% CI [0.99–2.78]) in PsA group. The aHRs of PsA were increased over time, aHRs was changed from 0.65 (0–11 months from index date) to 1.34 (≥12 months from index date) in all types of periodontitis and from 1.09 to 1.79 in moderate/severe periodontitis group, respectively.

**Conclusions:**

The increased risk of periodontitis was observed, especially the association between PsA and moderate/severe periodontitis. The patients with psoriatic disease should receive regular periodontal evaluation.

## Introduction

Psoriatic disease is an immune-mediated disease characterized by inflammation of the dermis and epidermis caused by atypical keratinocyte differentiation and hyperproliferation with predominantly skin and joint manifestations. The psoriasis (PS) patients may also have psoriatic arthritis (PsA) which cutaneous lesions indicative of PS precede development of arthritic signs and symptoms (Parisi et al., 2013; Wang, Hsieh & Tsai, 2016). The comorbidities reported to be associated with psoriatic disease include diabetes, metabolic syndrome, chronic obstructive pulmonary disease and cardiovascular disease (Tsai et al., 2011; Yang, Keller & Lin, 2011). It has been suggested that bacteria play an important role in the immunopathogenesis of psoriatic disease (Fry et al., 2013; Fry et al., 2015).

Periodontitis is a chronic inflammatory disease triggered by microbial biofilm and characterized by an immunologically moderated destruction of dental supporting tissues of the teeth, progressive attachment loss, and bone resorption, as well as characterized by pocket formation (Kinane & Bartold, 2007). The etiology of periodontitis relates to microbial biofilm infection and subsequent host-pathogen interactions. The association between periodontitis and immune-mediated inflammatory systemic diseases has been increasingly recognized (Ebersole et al., 2017).

Although psoriatic disease and periodontitis have same pathogenic mechanisms and associated conditions in common, few studies have shown that patients with periodontitis have a significantly elevated risk of PS (Keller & Lin, 2012; Nakib et al., 2013; Lazaridou et al., 2013; Skudutyte-Rysstad et al., 2014; Sharma, Raman & Pradeep, 2015; Egeberg et al., 2017). Contrarily, a few reports have shown no relationship (Fadel et al., 2013; Sezer et al., 2016) but not all studies have had negative findings in this regard.

However, the risk of periodontitis in patients with psoriatic disease still needs further investigation. The Taiwanese National Health Insurance Research Database (NHIRD) has facilitated many population-based longitudinal studies in Taiwan (Chen, Wu & Chang, 2017a; Chen, Wu & Chang, 2017b; Yang et al., 2017; Yang et al., in press). Therefore, this study assessed the risk of periodontitis in a large, nationally representative, population-based cohort of patients with psoriatic disease in Taiwan.

## Materials and Methods

### Database and ethical consideration

The NHIRD is a nationwide, single-payer, obligatory health insurance program launched on March 1, 1995, covering over 99% of the Taiwanese population in 2010 in Taiwan. The NHIRD is released for research purposes and consists of comprehensive claims data, including dental services, hospitalization services, outpatient services, traditional Chinese medical services, and detailed drug prescription records. To improve data accuracy, the Bureau of NHI routinely performs random checks of patient charts (Cheng, 2003). The Longitudinal Health Insurance Research Database 2010 (LHIRD 2010), a subset of NHIRD, was used for this cohort study. This LHIRD 2010 dataset included health care information from a randomly selected sample of one million beneficiaries in 2010. All subject information was anonymized and de-identified to protect privacy. On the basis of this detailed information, NHIRD can provide sufficient sample size, generalizability, and statistical power to assess epidemiological profiles of the entire Taiwanese population.

This study employed a retrospective cohort study design. The Ethics Review Board at the Chung Shan Medical University Hospital approved this study (CS2-15071). Informed consent was not required because this was a secondary data analysis. This report also complies with STROBE (Strengthening the Reporting of Observational Studies in Epidemiology) guidelines for observational studies.

### Exposure of psoriatic disease

The exposure group included patients who were newly diagnosed with psoriatic disease by the International Classification of Diseases, Ninth Clinical Modification (ICD-9-CM) codes 696.0 and 696.1 from 2003 to 2012. In order to avoid reverse causality, we excluded the prevalent cases with psoriatic diseases before 2003. Furthermore, the new psoriasis patients diagnosed in 2013 were excluded from analysis, because these patients were followed for less than one year. We excluded psoriatic disease patients without any treatment including the use of biologic drugs, corticosteroids, cyclosporine, psoralens, retinoids, methotrexate, or phototherapeutics within one year. The index date was identified as the date of initial psoriatic disease diagnosis. The psoriatic disease cases were classified into two groups, included PsA (ICD-9 code of 696.0) and PS (ICD-9 code of 696.1). If the case had both disease codes, we identified the case as PsA.

### Comparison group and propensity score match (PSM)

**Figure 1:**
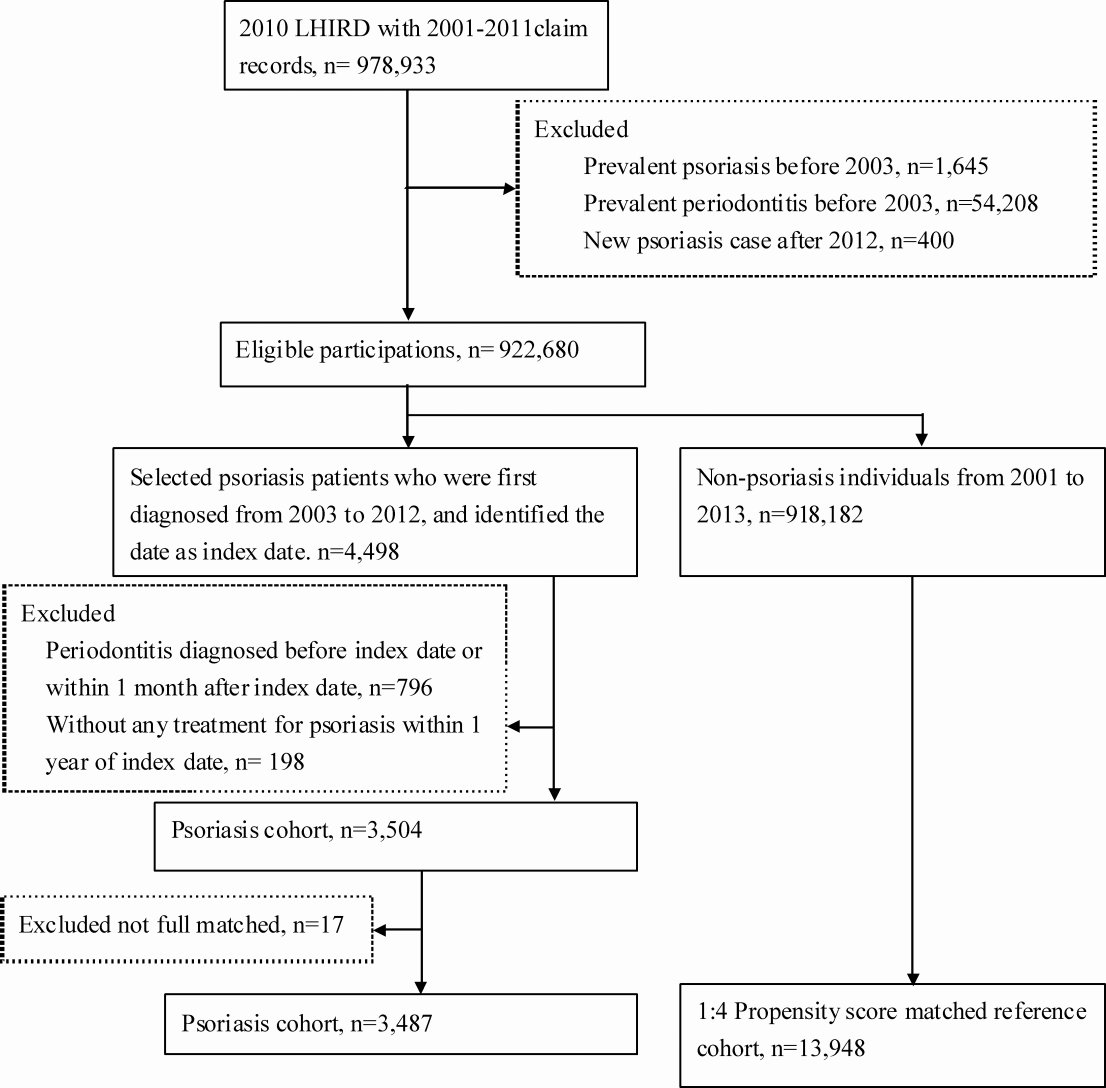
Flowchart of study design for propensity score matched retrospective cohort study.

As shown in Fig. 1, the comparison group included participations who were never diagnosed with psoriatic disease from 2001 to 2013. In order to reduce the confounding bias, we used propensity score matching to select controls. Propensity score of participants which predicted the probability of psoriatic disease exposure for participants was estimated by logistic regression modeling. The predictors involved birth year, sex, and co-morbidities at baseline. The 1:4 matched comparisons were selected with the same propensity score (stepwise selected from 8-digit exact matching to 1-digit exact matching) in as exposure subject. Furthermore, the controls were still at risk on index date.

### Periodontitis event

This study determined the occurrence of new cases of periodontitis by ICD-9 codes 523.3–523.5, which received ≥2 times dental scaling in 90 days or underwent periodontitis surgery. The severity of periodontal disease was classified into three levels included mild periodontitis (only dental scaling, ICD-9 procedure code 96.54), moderate periodontitis (subgingival curettage, ICD-9 procedure code 24.31), and severe periodontitis (periodontal flap operation, ICD-9 procedure code 24.39). The grade of the highest disease severity was identified within 90 days after periodontitis diagnosis.

### Co-morbidities

Potential confounders in this study were co-morbidities such as chronic obstructive obstructive pulmonary disease, COPD (ICD-9: 490, 491, 492, 494, and 496), diabetes (ICD-9: 250), hypertension (ICD-9: 401–405), and hyperlipidemia (ICD-9: 272). To improve the validity of the co-morbidities, these co-morbidities were identified as ≧2 outpatient visits or at least one admission within one year before index date.

The frequency of dental visit before index date was included in the analysis as the utilization of dental care also affected the probability of diagnosis with dental disease. Therefore, we used this variable as covariate in multiple regressions, or performed the stratified analysis by frequency of dental visit.

### Statistical analysis

This was a retrospective cohort study design with PSM, and we conducted a time to event analysis. All participations were followed from index date to event occurred or censored (death, withdraw, or the end of study on December, 31, 2013). The two tail *t* test and chi-square were used to test the difference of continuous and categorical variables, respectively. The generalized linear model with Poisson response and log function was applied to calculate the crude incidence rate of periodontitis and 95% confidence interval. The Cox proportional hazard models were applied to estimate the hazard ratios of periodontitis, and the multiple Cox models were conducted using age, sex and co-morbidities as covariates. All analyses were performed by SAS software (Version 9.4; SAS Institute, Inc., Cary, NC, USA), and the significant level was 0.05.

## Results

A total of 3,487 psoriatic patients diagnosed between 2003 and 2010 were included in the exposure cohort. The non-psoriatic disease group comprised 13,948 controls with 1:4 propensity score matched (Table 1). Patients with psoriatic disease were predominantly males (60.6% vs 39.4%) with a mean age of 45.28 years (standard deviation = 19.52). All baseline characteristics including the frequency of dental visit within 365 days prior to index date were not significantly different between psoriatic disease cohort and comparison. There were 415 (11.90%) PsA and 3,072 (88.10%) PS patients in psoriatic disease group.

**Table 1:** Demographic characteristics of study population.

	Comparison *n* = 13, 948	Psoriatic disease *n* = 3, 487	*p* value
Age	45.43 ± 19.62	45.28 ± 19.52	0.6782
Sex			0.4145
Female	5,391 (38.65%)	1,374 (39.40%)	
Male	8,557 (61.35%)	2,113 (60.60%)	
Co-morbidities			
COPD	486 (3.48%)	143 (4.10%)	0.0808
Diabetes	1,369 (9.82%)	336 (9.64%)	0.7499
Hypertension	2,658 (19.06%)	651 (18.67%)	0.6020
Hyperlipidemia	1,270 (9.11%)	311 (8.92%)	0.7317
Frequency of dental visit within 365 days prior to index date			0.2996
0	9,481 (67.97%)	2,313 (66.33%)	
1	1,707 (12.24%)	457 (13.11%)	
2	988 (7.08%)	259 (7.43%)	
≧3	1,772 (12.7%)	458 (13.13%)	
Type of psoriatic disease			
Psoriasis	–	3,072 (88.10%)	
Psoriatic arthritis	–	415 (11.90%)	

A total of 3,103 individuals with periodontitis were selected in this study. Patients (*n* = 345) were recognized as moderate/severe periodontitis among the periodontitis group. As shown in Table 2, crude incidence rates (per 103 person months) of periodontitis were 3.579 (95% CI [3.455–3.707]), 4.425 (95% CI [3.641–5.378]), and 3.652 (95% CI [3.393–3.932]) in comparison group, PsA, and PS group, respectively. For moderate/severe periodontitis, crude incidence rate (per 1,000 person months) were 0.398 (95% CI [0.358–0.442]), 0.657 (95% CI [0.396–1.090]), and 0.341 (95% CI [0.268-0.435]) in comparison group, PsA, and PS group, respectively.

**Table 2:** Incidence of periodontitis.

	Comparison	Psoriatic disease	
		Psoriasis arthritis	Psoriasis
	*n* = 13, 948	*n* = 415	*n* = 3, 072
Follow up person months	867,106	22,826	193,303
Periodontitis case			
All types	3,103	101	706
Moderate/severe	345	15	66
Incidence rate (per 10^3^ person months)			
All types	3.579 (3.455–3.707)	4.425 (3.641–5.378)	3.652 (3.393–3.932)
Moderate/severe	0.398 (0.358–0.442)	0.657 (0.396–1.090)	0.341 (0.268–0.435)

**Figure 2:**
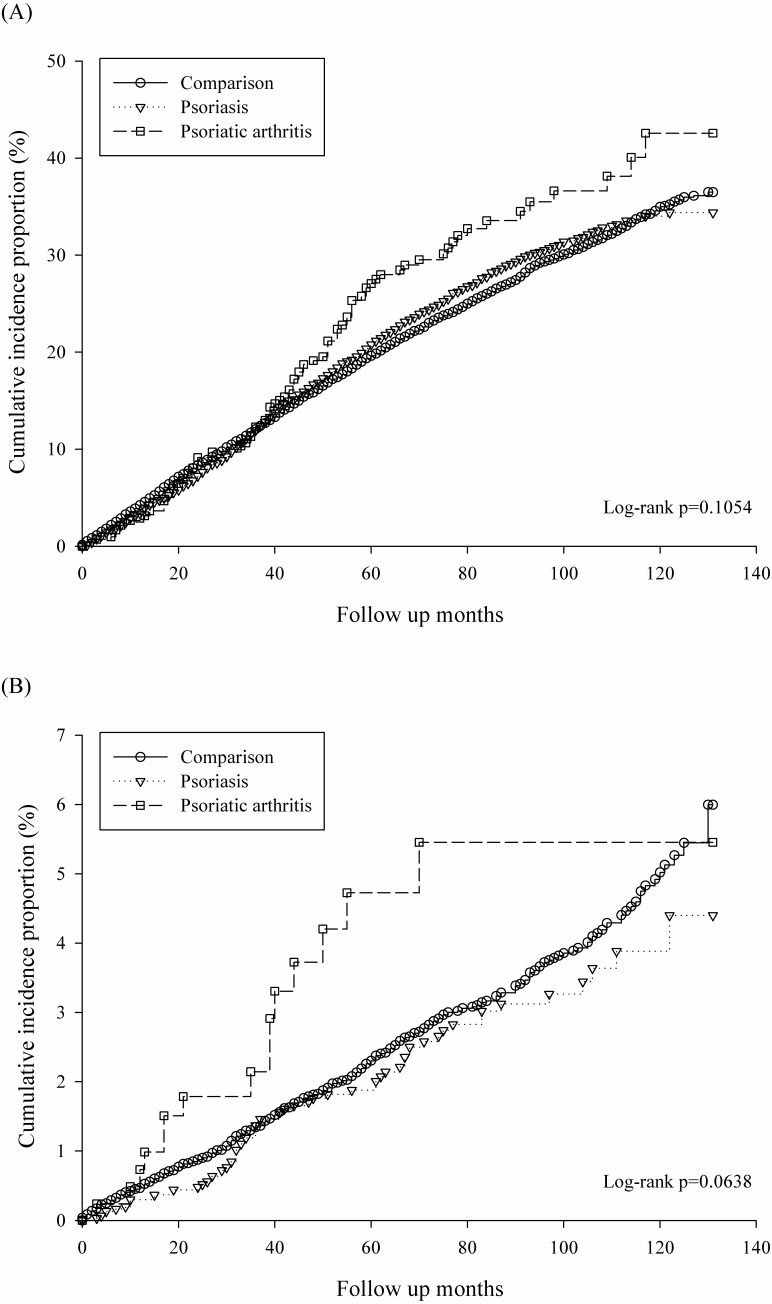
Kaplan–Meier curves of cumulative incidence proportion of periodontitis: (A) for all types of periodontitis, (B) for moderate/severe periodontitis.

Figure 2A revealed the un-adjusted Kaplan–Meier curves of cumulative incidence proportion in all types of periodontitis for psoriatic disease exposure. PsA group had higher risk of periodontitis, but the log rank test did not reach significance (*p* = 0.1054).In addition, Figure 2B demonstrated cumulative incidence proportion of moderate/severe periodontitis and PsA group had highest incidence risk of periodontitis with borderline significance (log rank *p* = 0.06).

The result of Multivariate Cox modeling is shown in Table 3. For all types of periodontitis, PsA had borderline significance for higher risk of periodontitis (adjusted HR, aHR = 1.20, 95% CI [0.99–1.47], *p* = 0.07) compared with comparison group. The risk of periodontitis was not significantly different between the PS group and the comparison group (aHR = 1.01, 95% CI [0.93–1.09], *p* = 0.84). For moderate/severe periodontitis, the aHRs were borderline significantly increased in the PsA group (aHR = 1.66, 95% CI [0.99–2.78], *p* = 0.06) and not significant in the PS group (95% CI [0.65–1.11], *p* = 0.22), respectively. Moreover, the pre-existence of COPD, hyperlipidemia and frequency of dental visit within 365 days before index date was significantly associated with periodontal treatment.

**Table 3:** Hazard ratios of periodontitis by multivariate Cox proportional model.

	All types of periodontitis		Moderate/severe periodontitis	
	aHR^a^ (95% CI)	*p* value	aHR^a^ (95% CI)	*p* value
Exposure group				
Comparison	Reference		Reference	
Psoriasis	1.01 (0.93–1.09)	0.8415	0.85 (0.65–1.11)	0.2233
Psoriatic arthritis	1.20 (0.99–1.47)	0.0681	1.66 (0.99–2.78)	0.0566
Age (per 1 year)	1.00 (1.00–1.00)	0.0531	1.01 (1.00–1.02)	0.0004
Sex				
Female	Reference		Reference	
Male	0.91 (0.85–0.97)	0.0033	0.98 (0.81–1.20)	0.8697
Co-morbidities				
COPD	1.25 (1.06–1.48)	0.0072	0.77 (0.44–1.34)	0.3555
Diabetes	0.95 (0.84–1.08)	0.4330	0.95 (0.68–1.33)	0.7583
Hypertension	0.98 (0.89–1.08)	0.7035	0.96 (0.73–1.26)	0.7819
Hyperlipidemia	1.22 (1.08–1.37)	0.0013	1.69 (1.24–2.29)	0.0008
Frequency of dental visit within 365 days prior to index date				
0	Reference		Reference	
1	1.57 (1.44–1.72)	<.0001	1.87 (1.44–2.42)	<.0001
2	1.69 (1.51–1.88)	<.0001	1.47 (1.03–2.09)	0.0350
≧3	1.83 (1.68–1.99)	<.0001	1.68 (1.29–2.19)	0.0001

**Notes.**

a Adjusted hazard ratio: this was adjusted for psoriasis exposure, age, sex, co-morbidities, and frequency of dental visit within 365 days prior to index date.

The results of further stratification by frequency of dental visit within 365 days prior to index date is shown in Table 4. The aHRs of moderate/severe periodontitis for PsA exposure were 1.87 (95% CI [0.96–3.66], *p* = 0.07) and 2.72 (95% CI [1.08–6.83], *p* = 0.03) in subgroup 1 (without dental visit) and 3 (received ≧ 3 times), respectively. However, there was no significnt association with periodontitis for exposure of PS in any subgroup stratified by dental care visits.

**Table 4:** The stratified analysis by the frequency of dental visit within 365 days prior to index date.

	All types of periodontitis		Moderate/severe periodontitis	
	aHR^a^ (95% CI)	*p* value	aHR^a^ (95% CI)	*p* value
Subgroup 1: without dental visit within 365 days prior to index date				
Comparison	Reference		Reference	
Psoriasis	1.08 (0.97–1.20)	0.1907	0.82 (0.58–1.17)	0.2782
Psoriatic arthritis	1.19 (0.90–1.57)	0.2334	1.87 (0.96–3.66)	0.0656
Subgroup 2: dental visit 1∼2 times within 365 days prior to index date				
Comparison	Reference		Reference	
Psoriasis	0.91 (0.77–1.08)	0.2728	1.05 (0.66–1.68)	0.8348
Psoriatic arthritis	1.19 (0.81–1.74)	0.3841	0.42 (0.06–2.98)	0.3818
Subgroup 3: dental visit ≧3 times within 365 days prior to index date				
Comparison	Reference		Reference	
Psoriasis	0.96 (0.79–1.17)	0.6910	0.58 (0.28–1.22)	0.1524
Psoriatic arthritis	1.29 (0.85–1.96)	0.2354	2.72 (1.08–6.83)	0.0334

**Notes.**

a Adjusted hazard ratio: this was adjusted for psoriasis exposure, age, sex, and co-morbidities.

**Table 5:** Hazard ratios of periodontitis stratified analysis by follow up period.^a^

	Hazard ratio (95% CI)			
	All types of periodontitis		Moderate/severe periodontitis	
	0–11 months from index date	≧12 months from index date	0–11 months from index date	≧12 months from index date
Exposure group				
Comparison	Reference	Reference	Reference	Reference
Psoriasis	0.80 (0.65–1.00)	1.05 (0.96–1.15)	0.66 (0.33–1.32)	0.89 (0.67–1.18)
Psoriatic arthritis	0.65 (0.36–1.19)	1.34 (1.09–1.65)	1.09 (0.27–4.47)	1.79 (1.03–3.13)

**Notes.**

a All models were adjusted for age, sex, co-morbidities, and frequency of dental visit within 365 days prior to index date.

Table 5 presented the time varied HRs of periodontitis for psoriatic disease exposure. For the early period (0–11 months from psoriatic disease diagnosed) of PS exposure, there was no significant association between PS and periodontitis. For the later period (≧12 months from psoriatic disease diagnosed), the results demonstrated at least one year exposure of PsA significantly increased the risk for all types of periodontitis (aHR = 1.34: 95% CI [1.09–1.65]) and moderate/severe periodontitis (aHR = 1.79: 95% CI [1.03–3.13]).

## Discussion

The relationship between psoriatic disease and periodontitis was first reported by Keller & Lin (2012) who stated the increased risk for PS among patients with chronic periodontitis in Taiwan. In this large-scale population-based longitudinal study, we first found that in Taiwanese patients with psoriatic disease, risk of periodontitis was increased compared to the general population and the incidence was highest for PsA. Similar results were found in a Danish nationwide cohort that the risk of periodontitis was highest in patients with severe PS and PsA (Egeberg et al., 2017). Moreover, we further classified the periodontitis based on the periodontal treatment records in this study. The patients with PsA had higher moderate/severe periodontitis probability than comparison group or PS group. This implies that the link between periodontitis and psoriatic disease depends on the disease severity.

In an age- and gender-balanced study of Turkish patients, demonstrated that periodontitis severity was higher in the PsA group (Üstün et al., 2013). Another study from a Norway University, in the propensity score (age, gender and education) matched sample (*n* = 100) psoriasis remained significantly associated with moderate/severe periodontitis (Skudutyte-Rysstad et al., 2014). Recently, a case control study showed that the periodontal status was associated with severity of PS in India (Sharma, Raman & Pradeep, 2015). However, these studies were all case-controlled in design and had limited sample sizes making it difficult to estimate the temporal frequency of periodontitis development in patients with psoriatic disease and generalize the study results.

To the best of our knowledge, our study is the first to evaluate the risk of periodontitis stratified by follow-up years in multivariable Cox proportional hazard regression. The risk of periodontitis was significantly higher in the PsA as compared to the PS group. This maybe because PsA is an advanced form of PS. Taken together, these findings indicate that intensive regular oral check-up for periodontal status is necessary for individuals with PS, especial for PsA patients.

The pathophysiology underlying the association between periodontitis and psoriatic disease remains hypothetical. The possible causes related to periodontitis in patients with psoriatic disease may include the underlying inflammatory and immunological processes of the diseases. It is well known that interleukin-17 plays a central role in innate immunity, inflammation and osteoclastogenesis all of which have been recognized in the pathogenesis of periodontitis as well as psoriasis (Zenobia & Hajishengalli, 2015). In addition, higher variety and concentrations of oral bacterial DNAs have been detected in synovial fluid compared to serum of PsA patients. In contrast, the predominant periodontal pathogens such as *T. forsythensis*, *P. gingivalis*, and *Prevotella species* were not detected in synovial fluids from patients with PsA (Moen et al., 2006). Bacteria originating from oral cavity might be important factors for the initiation and perpetuation of joint inflammation by oral microorganisms in PsA. However, this speculation still needs further investigation.

The Danish cohort study (Egeberg et al., 2017) used the Health Improvement Network which collected data only about hospital procedures (including hospital-based pharmacological treatment, e.g., with biological therapy) which are coded as treatment procedure codes (Andersen et al., 1999). The data source of current study was the NHIRD which collected data from both general practices and hospitals, allowing a more comprehensive data base and ultimately better generalizability of the findings.

This study still has potential limitations which should be considered. First, the data source of this study was the NHIRD which lacked relevant clinical variables such as laboratory data and pathology findings. In addition, the severity of periodontitis based on (ICD-9-CM) codes without correlated periodontal clinical parameters may not truly reflect the periodontal status. The codes for those patients who had osseous surgery, guided tissue regeneration, or implant due to severe periodontal disease, or extraction due to periodontal disease, could let these patients missed under “patient with periodontitis”. The operator variability in the treatment plan, the potential differences in patient acceptance of recommended treatment, patients’ compliance would also influence the data and hence the results of the study. There may be some patients with undiagnosed periodontitis in the control group. However, propensity score matching was performed in this study to reduce the selection bias and avoid the confounding variates. In addition, we identified the severity of periodontitis through the procedure codes for grading the specific periodontal condition. Third, the data on some potential factors for periodontitis and psoriatic disease, including tobacco use, physical activity, and body-mass index, were not available in the NHIRD dataset. Fourth, since this data is a claim-based of all medical treatments records among beneficiaries, the individuals served with more dental care had higher chance to be diagnosed with periodontitis. In this study, we added the frequency of dental visit within 365 days prior to index date to reduce the misclassification from information bias.

The results of this large-scale epidemiological study indicate that periodontitis is associated with increased risk of psoriatic diseases which was highest in patients with PsA. The mechanisms underlying this association require to be further clarified. Dentists should pay more attention to periodontitis patients with psoriatic disease.

## Conclusions

In this study, we found that the incidence rate of periodontitis were higher in patients with PsA. The aHRs of PsA were increased over time in periodontitis patients. As per the result section, majority of the associations noted were borderline and not statistically significant. Taken together, patients with psoriatic disease may benefit regular periodontal evaluation.

##  Supplemental Information

10.7717/peerj.4064/supp-1 Data S1 Raw data for analysis Observational raw data.

10.7717/peerj.4064/supp-2 Data S2 Code book for raw data Descriptions of variables in the raw dataset.

## Additional Information and Declarations

### Data Availability

The following information was supplied regarding data availability: The raw data has been provided in the Supplemental Files.
